# Advanced Materials in Cultural Heritage Conservation

**DOI:** 10.3390/molecules26133967

**Published:** 2021-06-29

**Authors:** Michele Baglioni, Giovanna Poggi, David Chelazzi, Piero Baglioni

**Affiliations:** Department of Chemistry and CSGI, University of Florence, Via della Lastruccia 3, 50019 Sesto Fiorentino, Italy; baglioni_michele@csgi.unifi.it (M.B.); giovanna.poggi@unifi.it (G.P.)

**Keywords:** advanced materials, gels, microemulsions, nanoparticles, functional materials, composites, Cultural Heritage preservation, cleaning, consolidation, protection

## Abstract

Cultural Heritage is a crucial socioeconomic resource; yet, recurring degradation processes endanger its preservation. Serendipitous approaches in restoration practice need to be replaced by systematically addressing conservation issues through the development of advanced materials for the preservation of the artifacts. In the last few decades, materials and colloid science have provided valid solutions to counteract degradation, and we report here the main highlights in the formulation and application of materials and methodologies for the cleaning, protection and consolidation of works of art. Several types of artifacts are addressed, from murals to canvas paintings, metal objects, and paper artworks, comprising both classic and modern/contemporary art. Systems, such as nanoparticles, gels, nanostructured cleaning fluids, composites, and other functional materials, are reviewed. Future perspectives are also commented, outlining open issues and trends in this challenging and exciting field.

## 1. Introduction

Cultural Heritage represents an invaluable socioeconomic resource. Works of art, when properly preserved and accessible, foster job creation, tourism, social inclusion, and cultural identity. Unfortunately, regardless of their diverse nature and composition, artifacts are inevitably exposed to recurring degradation processes. Environmental factors (temperature, light, relative humidity), anthropogenic causes (pollution, vandalism, wrong restoration interventions), biocontamination, natural disasters (floods, fires), and climate changes, all concur to threaten the preservation of our heritage and its transfer to future generations. Addressing such challenges has become a mandatory task, to which science has replied throughout the last decades by providing innovative materials and methodologies. Similar to medicine, with the artifacts in the role of the “patients”, scientific research has developed solutions for the diagnosis, preventive care, and remedial conservation of works of art. Much effort has been dedicated to diagnostic tools [1], where one of the latest applications involves the use of smart technology for monitoring the conservation state of the artifacts [2]. Besides, preventive measures have been described and implemented, aimed at minimizing the need for interventions [3,4]. However, artistic and historical objects will inevitably degrade, calling for the continuous development of advanced materials able to counteract specific degradation processes. The systematic formulation of tailored and functional materials to protect, preserve, and restore art represents an open challenge in research, replacing serendipitous approaches in the restoration practice.

In the following sections, we report the main highlights and advancements that occurred in the development of advanced materials for art conservation. The different systems are organized and classified according to their main purpose, ranging from cleaning to consolidation and surface protection. Diverse artistic substrates are addressed, including mural and canvas paintings, paper, wood, stone, and metals. Finally, future perspectives and open topics are discussed.

## 2. Advanced Materials for Cleaning

### 2.1. Cleaning—Definition and Traditional Approaches

Cleaning is one of the most common, delicate, and important operations in Cultural Heritage conservation. Besides restoring the aesthetic appearance of artifacts, cleaning has an active role in their preservation, as soiling and contaminating substances do not just alter readability, but also take part in, or induce, surface degradation processes, which may irreversibly alter the original components of works of art. A wide range of unwanted substances has to be routinely removed from artistic surfaces, including soil, common dirt, stains, soluble salts, aged materials applied either by the artist or during previous restoration interventions (adhesives, varnishes, protective coatings), alteration *patinae*, overpaintings, vandalism, etc. Thus, cleaning of artworks involves the removal of a number of different chemicals, spanning from inorganic salts, to small hydrophobic compounds, from natural polymers (proteins, polysaccharides, polyterpenes, etc.) to synthetic resins (acrylics, vinyl, etc.). A major requirement is that cleaning must be selective (i.e., only unwanted materials are to be removed) and non-harmful towards the artistic substrates. Additional requirements of the cleaning systems include eco-compatibility and reduced toxicity, so as to address health concerns related to the environment and the operators.

In the past, conservators and restorers mostly used a trial-and-error approach, and employed as cleaning tools a wide palette of natural and common materials, as biological fluids (saliva, bile, or even urine), edible ingredients (wine, garlic, bread, and hot oil), or ash, and rudimental soaps [5]. Some of these materials indeed contain surfactants, enzymes, and chelating agents, which are similar to modern chemicals used in most of the current advanced cleaning formulations. However, cleaning materials specifically designed for the conservation of Cultural Heritage were developed only in the 1980s–1990s [5,6]. Up to the early XX century, the removal of soil or generic unwanted materials was mainly carried out using organic solvents, sometimes thickened with natural or synthetic polymers. This traditional approach is still widely used, and mostly relies on the evaluation of solubility parameters, such as those proposed and defined by Hildebrand [7,8,9] and later modified and simplified by Teas [10], or, more recently, to solvatochromic properties and equilibrium swelling capacity [9,11], which are used to select solvent blends to swell or solubilize the unwanted compounds. However, this traditional approach presents significant drawbacks, i.e., the lack of safety to artworks, poor eco-compatibility, and non-negligible toxicity of the solvents. The latter are usually applied either as non-confined (using cotton swabs or brushes) or poorly confined as thickened pastes using polymers, such as cellulose ethers or polyacrylic acid [5,12,13]. This approach is hardly selective and poorly controllable, generating major issues, such as the scarce temporal and spatial control of the cleaning action, and the possible presence of polymer residues, which may need invasive rinsing steps. Most recently, the contribution of nanoscience and materials science to the field of preservation of Cultural Heritage, allowed overcoming these limitations, and provided the conservators’ community with a palette of advanced systems and methodologies that are revolutionizing the cleaning of works of art [6,14,15]. 

### 2.2. Nanostructured Fluids

Nanostructured fluids (NSFs) are colloidal systems based on the peculiar properties of surfactants. Above a critical concentration, surfactant molecules in water self-assemble to form *micelles*, i.e., nanosized supramolecular aggregates, whose hydrophobic core may act as solubilization sites for small hydrophobic molecules. This feature is the basis of detergent properties of surfactant solutions. The addition of one or more organic solvents to aqueous surfactant solutions generates a variety of different colloidal systems, which differ according to the chemical nature of the components and their relative amounts. Oil-in-water (o/w) microemulsions are thermodynamically stable fluids composed of nanosized droplets of water-immiscible organic solvents dispersed in an aqueous continuous phase by a palisade of surfactant molecules (and often co-surfactants, such as medium-length-chain alcohols). Typically, NSFs include 60–90% (*w*/*w*) of water, which greatly decreases their environmental impact and toxicity, as opposed to traditional solvent blends [16,17,18,19].

The first application of a NSF, an o/w microemulsion, to conserve Cultural Heritage tracks back to 1986 and represents a milestone for the development of advanced cleaning systems for artworks: an aqueous cleaning fluid including n-dodecane, n-pentanol, and an anionic surfactant (sodium dodecylsulfate, SDS) was used for the first time to effectively solubilize and remove detrimental wax stains from the surface of Renaissance frescos in Florence, Italy [20]. Since then, several different NSFs were developed and applied to art conservation as novel cleaning systems for the removal of a variety of unwanted materials (soil, small apolar molecules, varnishes, synthetic polymeric coatings). 

**Figure 1:**
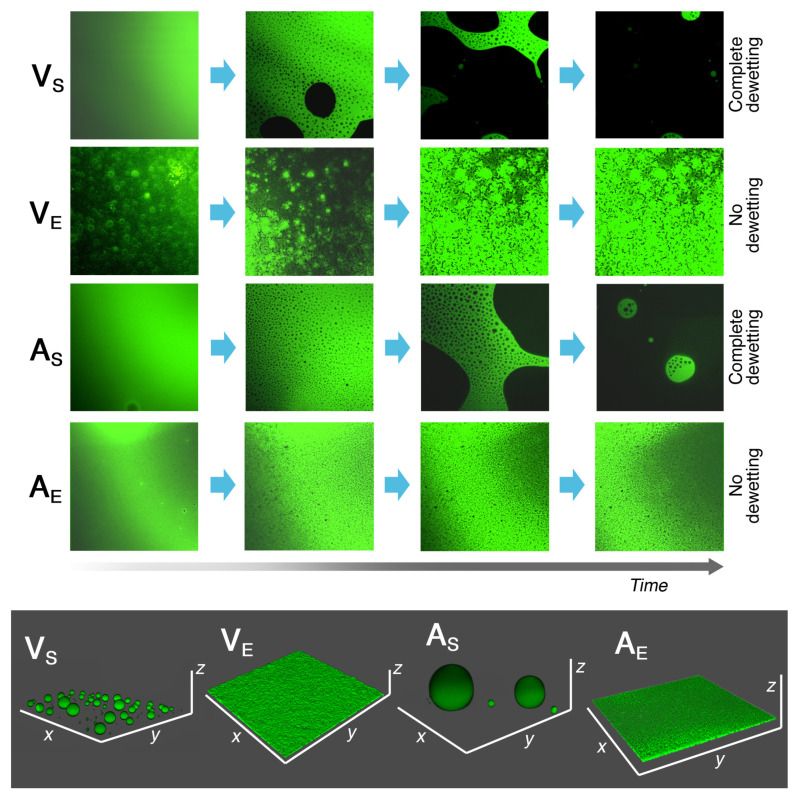
Confocal laser scanning microscopy imaging experiments on 2–4-μm-thick polymer films cast on glass slides. The films were incubated with a NSF, and their morphology changes were followed over time. Vinyl and acrylic polymers cast from solvents’ solutions (V_S_ and A_S_) are completely dewetted, while the same polymers cast from aqueous emulsions (V_E_ and A_E_) are still intact after 10 min of incubation with the NSF. Bottom box: 3D reconstruction of the four polymers’ morphology after 10 min of interaction with the NSF. Reprinted from Reference [28], Copyright (2019), Baglioni, Alterini, Chelazzi, Giorgi, and Baglioni.

More recently, some of the main aspects about the nanostructure of NSFs [18,21,22], as well as the physico-chemical processes underpinning their cleaning effectiveness [23,24,25,26,27,28,29], have been unveiled. The solubilization of apolar molecules inside the hydrophobic core of micelles is responsible for the detergency properties of NSFs, and drives the removal of low molecular weight chemicals (fatty acids, triglycerides, hydrocarbons, which are common constituent of soil and grime) [30,31]. Other phenomena regulate the interaction of NSFs with polymeric films, such as adhesives, protective coatings, consolidants, and varnishes frequently found on artistic surfaces. It was shown that a dewetting process can occur when a hydrophobic polymeric coating is removed from a hydrophilic solid surface (plaster, stone, metal, glass) using water-based NSFs, which, as a whole, are non-solvents for the film [24,25]. In particular, polymer films cast from solvents’ solutions tend to dewet, while polymer latexes cast from aqueous emulsions can be swollen and softened by the action of NSFs, mainly due to the significant presence of amphiphilic additives in the film (see Figure 1) [28]. The solvents and surfactants in the NSFs interact synergistically and, in certain conditions, can induce dewetting. Organic solvents swell and soften the film, enhancing the mobility of macromolecular chains (with a subsequent lowering of the polymer glass transition temperature). Surfactants, on the other hand, have a twofold role: (i) decrease interfacial energies, and, consequently, the energetic cost to initiate dewetting; and (ii) penetrate into the film [27], possibly contributing to further lowering the glass transition temperature. It was demonstrated that nonionic surfactants (e.g., alcohol ethoxylates) are more efficient than ionic amphiphiles [25] in inducing dewetting; hydrophobicity and molecular conformation can further increase this trend, as shown for methyl-capped ester ethoxylates with respect to ordinary alcohol ethoxylates [31]. The main advantages produced by this interaction mechanism, as opposed to traditional solvents chemistry, are evident: in the latter case, solvents or solvent blends dissolve the coating and spread it deeper into the artifact porosity, while NSFs are able to dewet, or swell, and detach polymer films, rather than solubilizing them, allowing for their complete and safe removal [25].

Aqueous NSFs have been assessed in a vast number of case studies during the last few decades. They were tested in the removal of detrimental and aged polymeric coatings, applied during past conservation interventions or vandalism, from Renaissance and Middle Age mural paintings, Mesoamerican and Middle East archaeological sites [14,15,32]. It is known that polymeric coatings alter the interfacial properties of stone and wall paintings [33], promoting the damage caused by soluble salts normally present in masonry. Moreover, the polymers themselves can degrade, adding further damage to the surface of the works of art. Several different formulations have been developed over the years, including “green” and non-toxic solvents, such as alkyl carbonates [18,34,35], and readily biodegradable [22,31] or self-cleavable surfactants [15]. This latter class of amphiphiles further lowers the impact of NSFs on the environment, besides reducing the amount of non-volatile residues left on the treated artwork surfaces [36].

The use of water-in-oil (*w*/*o*) microemulsions for cleaning of water-sensitive artistic surfaces was also recently proposed and discussed [37,38,39]. *w*/*o* microemulsions are colloidal systems where nanosized water droplets are stabilized in a continuous phase of organic solvents by the presence of one or more surfactants. The reason behind the application of these systems to art cleaning is to control the delivery of aqueous solutions onto water-sensitive surfaces by lowering the water content and confining water droplets in an apolar solvent. Usually, hydrocarbons or low-molecular weight cyclosilicones are employed to this aim, in view of their inertness to modern paint layers, such as acrylic paint or modern oils. The dispersed water phase, possibly containing chelators or pH buffers, combined with the effect of surfactants, should be effective in the removal of hydrophilic dirt [40]. Some interesting applications of this approach have been reported; however, possible drawbacks are due to: (i) the use of significant amounts of surfactants, needed to stabilize water droplets in apolar solvents, which can remain as possibly harmful residues on artistic surfaces; and (ii) the fact that cyclosilicones have been recently identified as toxic compounds.

**Figure 2:**
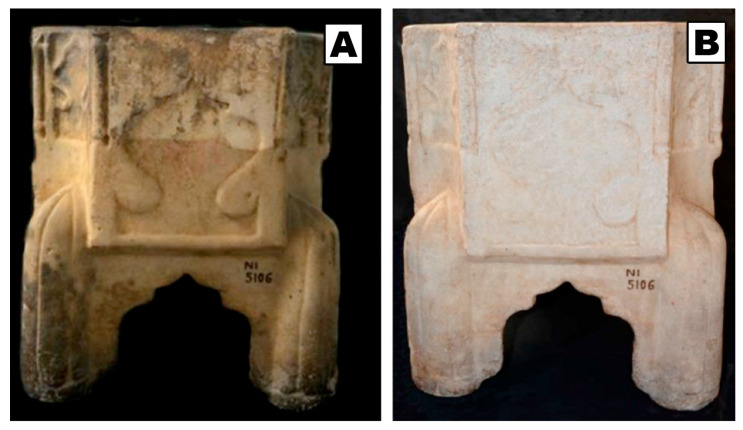
SDS-modified halloysites were used to formulate inorganic o/w emulsions with hydrocarbons and effectively used to clean marble artifacts. The picture shows the artifact before (**A**) and after (**B**) the application of the oil-in-water emulsion based on SDS/halloysite inorganic micelles and tetradecane. Adapted with permission from Reference [41], Copyright (2020), American Chemical Society. Further permissions related to the material excerpted should be directed to the American Chemical Society.

Most recently, new nanostructured fluids were investigated, such as inorganic and pickering emulsions [41], which build on the pathways investigated in last decades, to explore new possibilities. In particular, halloysite nanotubes modified with SDS were proposed to formulate *o*/*w* emulsions, which were efficiently used for the cleaning of marble artifacts (see Figure 2) [41,42].

### 2.3. Gels and Polymer Networks

Control on the cleaning of sensitive surfaces can also be achieved by confining liquid systems into gels and highly viscous polymeric dispersions (HVPDs) [5,14]. As said above, thickeners have been traditionally used in conservation to increase the viscosity of solvents and aqueous solutions. However, thickened systems are prone to leave residues on treated surfaces, and provide only poor control on the cleaning action, two major issues. Rigid polysaccharides gels, such as gellan or agarose [5], more recently introduced in the restoration practice, cope with the residue issue but are unable to adapt to the irregular surfaces commonly found in modern and contemporary art [43,44,45].

To overcome these limitations, a variety of advanced gelled materials based on chemical (held by covalent bonds) or physical networks (held by secondary bonds) were recently proposed [14]. Two main features are required in this sense: (i) gels or gel-like networks must have high viscoelasticity, in order to favor handling, effective and homogeneous adhesion, and, most importantly, the capacity to be easily and completely removed in one piece without the necessity of rinsing steps to eliminate possible residues; and (ii) they must be able to upload large amounts of water or solvents, and release them at highly controlled rate. The rationale underpinning most of the proposed synthetic pathways is the possibility to adjust and tune parameters, such as pores’ size distribution, polymer average mesh size, mechanical properties (i.e., elastic and viscous moduli), free water index, diffusivity of fluids inside the network, and water/solvent release rate.

**Figure 3:**
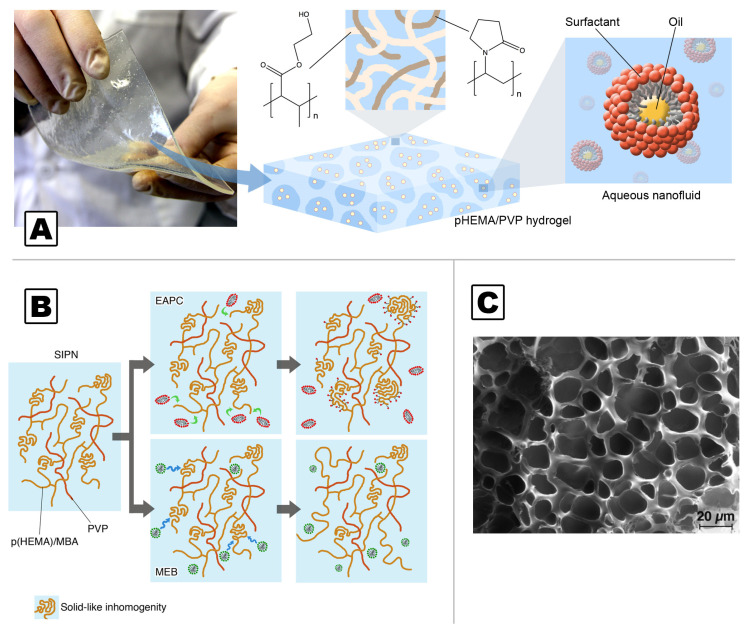
NSFs can be loaded into pHEMA/PVP SIPNs, which are usually synthesized in the form of flexible sheets. Pores’ size allows the diffusion of supramolecular aggregates into the gel matrix without significant alteration of either the gel or the NSF (**A**). Reprinted from ref [47] by permission from Springer, Analytical and Bioanalytical Chemistry, Nanofluids and chemical highly retentive hydrogels for controlled and selective removal of overpaintings and undesired graffiti from street art, R. Giorgi, M. Baglioni, P. Baglioni, Copyright (2017). Depending on surfactants’ chemical nature, the polymer network can be slightly modified; ionic surfactants tend to adsorb onto solid-like domains, while nonionics are able to migrate and swell solid-like polymeric blobs. As a whole, however, the mechanical and rheological properties of the gel are maintained (**B**). Scanning electron microscopy image of a freeze-dried pHEMA/PVP SIPN, showing a micrometric open porosity, which allows effective diffusion of the micelles (**C**). (**B**,**C**) Adapted with permission from Reference [48], Copyright (2018), American Chemical Society. Further permissions related to the material excerpted should be directed to the American Chemical Society.

A recently proposed class of chemical gels for art cleaning is based on chemical networks of poly(hydroxyethyl methacrylate) semi-interpenetrated with linear chains of polyvinylpyrrolidone (pHEMA/PVP SIPNs). These viscoelastic hydrogels are highly retentive, and can be loaded with aqueous solutions or o/w NSFs (see Figure 3) to selectively remove unwanted layers from water-sensitive surfaces in a controlled and safe way [44]. These SIPNs, typically synthesized in the form of 2-mm-thick sheets, are easily handled and removed after their application. They have been successfully used to clean fragile canvas paintings, murals, and paper artifacts [6,14,44,46].

Another class of retentive gelled systems for art cleaning includes dispersions and gels based on polyvinyl alcohol (PVA), which is a green, sustainable, and biocompatible polymer. PVA is capable of forming 3D networks via different synthetic pathways, such as cast-drying, freeze-thawing, and chemical manipulation [45,49,50,51]. PVA-based systems were introduced in art conservation at the beginning of the 2000s, when HVPDs based on borate/PVA interactions were used to confine aqueous solutions and hydro-alcoholic mixtures. The viscous dispersions could be easily peeled off the surface after their application, leaving no residues, which represented a significant advancement with respect to traditional thickeners [14,49,52,53,54]. Even if these HVPDs were assessed in several case studies with good results, their development was later abandoned, in view of possible health concerns posed by borate, and alternative PVA-based formulations were more recently considered. A class of PVA-based hydrogels was obtained by freeze-thawing cycles, which are able to create physical networks of PVA crystallites, holding together polymer chains, without the addition of a cross-linker. Different properties can be conferred to the PVA 3D network by including in the formulation either PVP or a PVA with different molecular weight and hydrolysis degree [45,50]. The so-obtained new class of gels, namely PVA/PVA “twin-chain” networks, was developed and effectively used to remove surface soiling and aged varnishes from Picasso, Pollock, and Lichtenstein’s paintings [45,55,56]. The inclusion of the low-molecular weight PVA allows obtaining a network with a sponge-like interconnected porosity, which is significantly different from the pores of the single-PVA homologue gel. This difference is likely to play a key role in the absorption of dirt from surfaces, due to the interconnected pores that capture soiling particles and favor their migration through the gel network. Compared to the single-PVA gel, the “twin-chain” PVA network also adapts more homogeneously to irregular and textured surfaces, which makes it particularly suited for modern and contemporary paintings [45]. 

**Figure 4:**
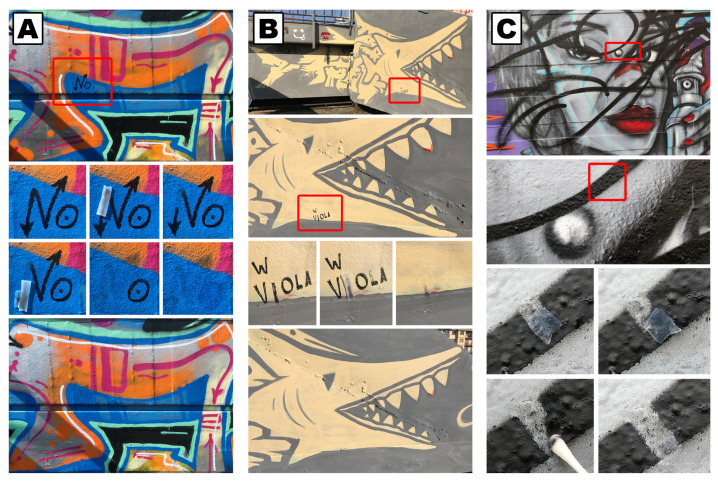
Complete removal of black tags realized with felt-tip permanent markers on street art, using NSF-loaded highly retentive hydrogels (**A**,**B**). Preliminary tests on the removal of vandalizing spray-can black tags realized on a figurative street art piece (**C**). The action of the NSF-loaded hydrogel is visible in the close-up pictures. The black paint layer is selectively swollen and softened, and subsequently removed via a careful and gentle mechanical action performed with humid cotton swabs. Adapted from Reference [35], Copyright (2021), with permission from Elsevier.

Similar to pHEMA/PVP, also PVA-based hydrogels can be loaded with aqueous NSFs, thanks to the ability of the micelles to diffuse through the polymeric network without a significant alteration of either their structure or the gel’s [46,48,55]. The combination of NSFs and highly retentive hydrogels represent one of the most advanced and effective cleaning solutions today available to restorers and conservators. NSF-loaded hydrogels can be used to highly enhance control of the cleaning action on extremely water-sensitive surfaces. Moreover, they offer a unique solution to the highly challenging issue of selectively removing overpaintings, which are commonly found, for instance, as vandalism on street art (see Figure 4) [35,47].

Finally, opportunely formulated PVA dispersions can also be cast directly on the surface of artworks, let dry until film formation, and then peeled off the surface, removing stubborn dirt or other unwanted materials. This method has been used for the removal of corrosion and alteration *patinae* from historical bronze, using chelating agents confined in the PVA dispersion [57].

### 2.4. Biocleaning

Starting from the 2000s, biotechnologies contributed to the research and development of promising and advanced alternatives to traditional methodologies for the cleaning of artistic surfaces. It was demonstrated that microorganisms and their metabolic processes could be effectively used for conservation [58]. All the proposed methods and cleaning systems go under the generic term of *biocleaning*. This field has encountered growing interest, mostly because it is based on eco-friendly and safe procedures, obtained by the optimization of natural and biological reactions in microorganisms’ metabolism, all aimed to art conservation. At present, biocleaning represents an interesting alternative to traditional cleaning methodologies [59,60], and, during the last 20 years, several papers reported on case studies where biotechnology was applied to art conservation. In particular, these included the removal of nitrates and sulfates from stone, the elimination of black crusts [61], and the removal of aged organic compounds from frescos [59]. However, even if the number of scientific papers on this topic has been growing, the availability of ready-to-use cleaning systems and their employability on a large scale remain challenging issues [58].

## 3. Advanced Materials for Consolidation

Cultural Heritage materials usually display a significant worsening of their mechanical properties as a result of several degradation processes that occur upon natural aging. Just to mention the most recurring cases that will be discussed in the following paragraphs, these processes may result in: (i) flaking and powdering of pictorial layers; (ii) blistering, delamination, and cracks in mortars, stones, and cements; and (iii) weakening and tearing of natural fibers.

### 3.1. Consolidation of Carbonate-Based Works of Art

The consolidation of flaking murals’ surfaces was the application that paved the way for the development of advanced materials belonging to colloids and materials science for the conservation of works of art. As a consequence of the flood of the Arno river, which took place in Florence in 1966, a wide number of artworks were contaminated and endangered by salts and detrimental organic compounds. 

With the aim of extracting sulfates from weakened *frescoes*, and then gaining back the original cohesion of the pictorial layer, Enzo Ferroni, a physical-chemist of the University of Florence, proposed the use of ammonium carbonate and barium hydroxide aqueous solutions, developing a method that refashioned the traditional approach in the consolidation of *frescos* [62]. In fact, differently from the polymeric adhesives commonly used in the 1960s to reattach flaking parts, Ferroni’s method was based on chemicals that were totally compatible with the original substrates from a physico-chemical standpoint. In other terms, rather than covering the surface of frescoes with a polymeric coating that would completely alter the porosity and vapor permeability of the paint layer [62], Ferroni consolidated the murals by re-creating lime, the original binder, that set into calcium carbonate due to reaction with atmospheric CO_2_, as it normally happens during the realization of *frescos*.

**Figure 5:**
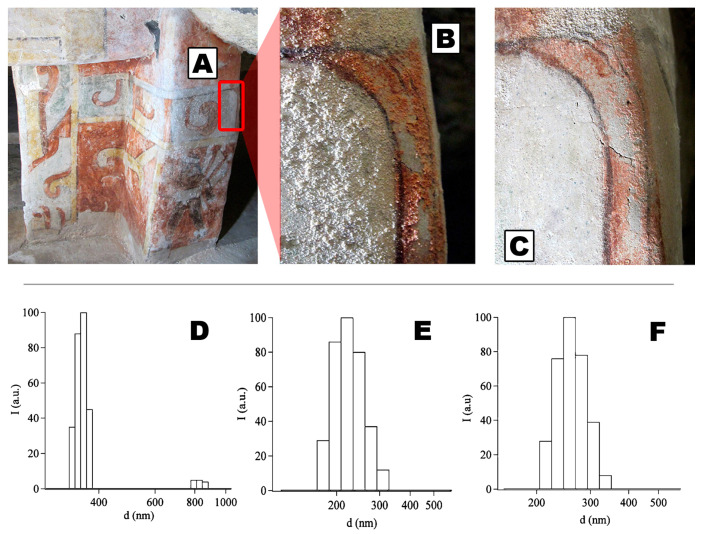
A degraded wall painting belonging to a Mesoamerican archeological site (**A**), exhibiting sulfate efflorescence (**B**). The sulfates were removed by treatment with ammonium carbonate and, afterwards, the surface was consolidated with calcium and barium hydroxide nanoparticle dispersions (**C**). Size distributions of Ca(OH)_2_ nanoparticles in 2-propanol (**D**), Mg(OH)_2_ nanoparticles in 2-propanol (**E**), and Ba(OH)_2_ nanoparticles in 1-propanol (**F**) designed for use in Cultural Heritage conservation. Adapted with permission from Reference [62], Copyright (2013), American Chemical Society. Further permissions related to the material excerpted should be directed to the American Chemical Society.

Building on this alternative approach, the following step forward in the consolidation of degraded carbonate-based materials was the formulation of Ca(OH)_2_ nanoparticle dispersions in short chain alcohols, which dates back to the late 1990s (see Figure 5) [63,64]. 

Here, the consolidating effect is directly due to the transformation of highly crystalline platelets of Portlandite, approximately 100–200 nm wide and a few nm thick, into calcium carbonate upon reaction with CO_2_, after solvent evaporation. The use of short chain alcohols, instead of water, allowed to increase the amount of active material delivered into the degraded artworks and avoided possible alterations of alkaline-sensitive pigments and binders. Different synthetic strategies have been tested over the years, including bottom-up and top-down procedures, resulting in nanoparticles having different size distribution and physico-chemical properties [65,66]. 

Besides the development of different synthetic pathways of calcium hydroxide nanoparticles for stones and mortar consolidation, research efforts have also been devoted to clarifying the mechanisms involved in carbonation reactions. In this regard, several studies have been published over the years about the kinetics of Ca(OH)_2_ nanoparticles conversions into CaCO_3_ as a function of the environmental conditions and of the physico-chemical properties of the reacting particles, with the aim of completing the picture about carbonation process in Cultural Heritage consolidation [67,68,69,70,71].

### 3.2. Consolidation of Earthen Materials

The majority of historical buildings and archaeological remains made of earthen materials usually exhibits damages and mechanical failures, making their consolidation an urgent task in conservation practice. Given the importance of this type of building materials, widely used in underdeveloped countries and alternative to cements (which are known to contribute to global warming), practical solutions for the consolidation of earthen masonry represent an application with high potential socio-economic impact.

Unfortunately, apart from the use of synthetic polymers, which show poor or no compatibility with the original materials, only few solutions have been proposed for the consolidation of earthen masonry, i.e., alkaline activation of bulk silica with aqueous solutions of hydroxides [72]. 

One of the most promising development is based on the use of calcium hydroxide and silica nanoparticles stably dispersed in a hydro-alcoholic mixture in the presence of a cellulose ether [73]. The reaction between hydroxide and silica nanoparticles allows the formation of calcium silicate hydrate (CSH), as it commonly happens during the settling of Portland cement, providing consolidation to crumbling adobe, which then shows enhanced resistance to peeling, abrasion, and wet-dry cycles.

### 3.3. Concrete Consolidation

**Figure 6:**
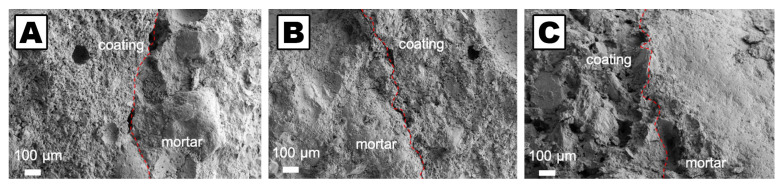
Scanning electron microscopy pictures showing the interphases (red dotted lines) between treated and untreated mortars’ portions. Interphases can be hardly seen due to the fact that the used consolidants for detachments (**A**), for small cracks (**B**), and for big cracks (**C**), did not altered the morphology and compactness of the samples. Adapted from Reference [74], Copyright (2021), with permission from Elsevier.

The restoration of concrete-based materials is an emerging research field, given the number of historical monuments built in the last century using concrete that are in need of conservation interventions. To address the damages that affect these structures, two different approaches have been recently explored: (i) cement formulations enriched with inorganic admixtures, namely silica and benzotriazole-loaded halloysites, have been used to recover small damages, such as delaminations and cracks (<1 cm), and, at the same time, to address in an innovative way the problem of corrosion of steel rebars (see Figure 6) [74]; and (ii) silica oligomer based-impregnation treatments have been used to produce a C-S-H gel from the reaction between silanol groups from the silica-based products and Ca^2+^ ions from original portlandite, resulting in a simple, compatible, and effective consolidation of degraded cements [75].

### 3.4. Strengthening and Deacidification of Fibrous Materials

Acid-catalyzed degradation reactions commonly affect cellulose- and collagen-based fibrous artifacts, resulting in a decrease of the pH and of the original mechanical resistance. In that sense, deacidification, i.e., the neutralization of acidity in the artistic material, and strengthening treatments are key steps in the remediation process that secures the preservation of these heritage materials. Over the years, several methods have been proposed for the deacidification of cellulose-based objects [76,77]. In particular, colloidal earth-alkaline hydroxides and carbonates in organic solvents have been employed to adjust the pH of cellulose- and collagen-based artifacts (paper, wood, parchment, and leather) [78,79,80,81]. The beneficial effect of alkaline nanoparticles for the preservation of acidic paper has been also recently shown by NMR diffusometry and relaxometry measurements [82,83].

Starting from these applications, different types of advanced materials have been proposed, alone or combined in hybrid systems, for the pH-adjustment and/or the strengthening of different types of fibrous substrates.

For instance, colloidal dispersions of Mg(OH)_2_ nanoparticles stabilized by trimethylsilyl cellulose (TMSC) and dispersed in hexamethyldisiloxane have been recently proposed for the concomitant deacidification and strengthening of paper substrates. While alkaline nanoparticles buffer acidity, TMSC, upon aging, is hydrolyzed into cellulose inducing an increase of about 20% in the tensile strength of the treated paper [84]. Hybrids of oleic acid-grafted cellulose nanocrystals and alkaline nanoparticles have been recently proposed for the concomitant pH-adjustment and strengthening of degraded paper samples. The grafting of nanocellulose with oleic acid allowed the formulation of stable dispersions in ethanol, a solvent that can be safely used on water-sensitive works of art, which can also be mixed with CaCO_3_ and Ca(OH)_2_ nanoparticles obtained from a solvothermal reaction [85]. It is worth noting that these mixtures are thixotropic, which allows their application as fluids, or, when a more confined application is required, in a gel-like state. The application of these systems on degraded paper improved the mechanical properties and adjusted pH.

**Figure 7:**
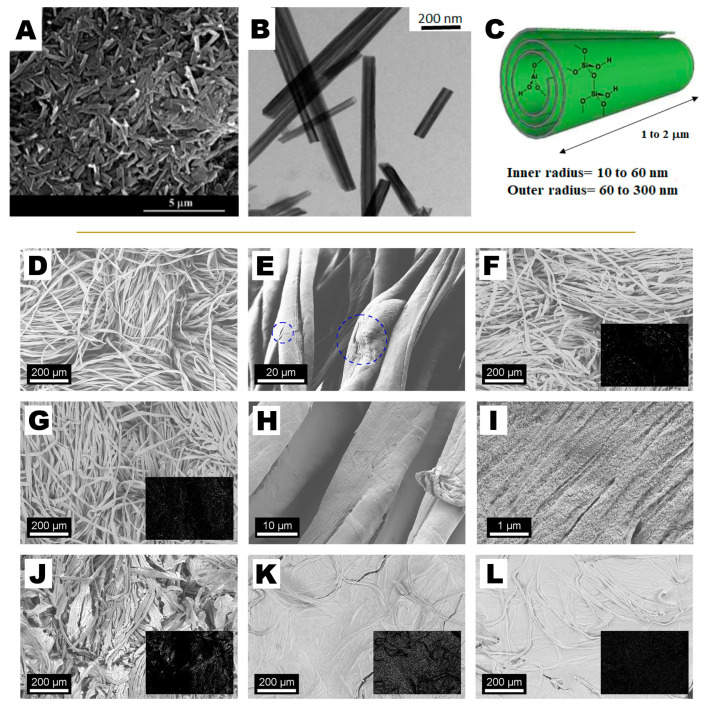
Scanning electron microscopy (**A**) and Transmission electron microscopy (**B**) images of halloysite nanotubes. Schematic representation of the spiral-like morphology of halloysite used for consolidation and deacidification of waterlogged wood (**C**). Scanning electron microscopy images of degraded cotton canvas before (**D**,**E**) and after treatment with SNP and CNF (**F**–**L**). The insets show the abundance of silica on the surface of the samples as obtained from Energy-dispersive X-ray spectroscopy. (**A**–**C**) Adapted with permission from Reference [41], Copyright (2021), American Chemical Society. Further permissions related to the material excerpted should be directed to the American Chemical Society. (**D**–**L**) Adapted with permission from Reference [89], Copyright (2018), American Chemical Society. Further permissions related to the material excerpted should be directed to the American Chemical Society.

The consolidation of waterlogged archaeological wood is a challenging operation, which still needs to be successfully tackled by scientists and conservators. To this aim, Cavallaro et al. have formulated nanohybrids featuring polyethylene glycol, a traditional consolidant, and functionalized halloysite nanotubes filled with Ca(OH)_2_ (see Figure 7A–C). Gradual release of the alkalis inside the treated samples allowed neutralization of acidity, while the nanohybrids act as a strengthening agent, penetrating the wooden matrix and increasing its flexural strength and rigidity [41,86].

A different approach for the consolidation of degraded wood, making use of nano-scale consolidants solely based on cellulose, has been recently proposed [87]. Specifically, cellulose nanocrystals have been mixed with hydroxypropyl cellulose to achieve efficient consolidating action. The addition of cellulose nanocrystals increased the penetration of the consolidant within the wood matrix, reduced the color change induced by the application, and increased the compression strength of the treated samples.

Mixtures of polyelectrolyte-treated silica nanoparticles (SNP) [88] and cellulose nanofibrils (CNFs) have been used to treat the backside canvases of classic and modern paintings [89,90] or to address the conservation of iron-tannate dyed cotton [91]. The main strengthening effect induced by the presence of silica particles is an increase in the fiber stiffness, most likely owing to dense packing of the particles at the fiber surface, while CNFs film at the treated surface, allowing for increased ductility of the canvas. The two effects can be balanced by varying the SNP/CNFs ratio, resulting in an effective consolidating effect (see Figure 7D–L). These materials can be also combined with alkaline compounds to mechanically stabilize the original material and to counteract the acidity that promotes degradation.

Finally, one of the latest applications addresses alterations in the secondary structure of fibroin in silk artifacts, which weaken the mechanical properties of degraded textiles, whose conservation is a challenging operation. The use of self-regenerated silk fibroin, prepared from waste silk, has been recently proposed as a green, effective and compatible consolidant for historical artifacts [92]. The application of fibroin with different degrees of amorphousness or crystallinity, obtained from diluted or concentrated protein dispersions, results in changes in the mechanical properties of the treated silk fibers, which show brittleness in the presence of crystalline layers and ductility when amorphous fibroin is applied.

## 4. Advanced Materials for Surface Protection

The protection of artistic surfaces is one of the oldest practices in restoration. However, as discussed in the previous sections, traditional varnishes and coatings undergo aging, and can even promote the alteration and degradation of works of art. Looking for alternative materials for protecting surfaces against dirt, erosion and corrosion, research has developed a series of innovative formulations over the last decades. One common trend is the combination of inorganic nanomaterials into polymer matrices, to obtain enhanced properties. For instance, halloysite and other nanoclays have been recently incorporated into polymeric matrices to form protective coatings that prevent the degradation of surfaces induced by atmospheric pollutants, corrosive agents and entomological attacks [93]. In the following sections, we report some of the most interesting results regarding the protection of stone and metal artifacts.

### 4.1. Surface Protection of Stones and Buildings 

One of the most recurring issues in the conservation of outdoor artistic and architectural heritage is biodeterioration, which can produce permanent alteration of surfaces. In this regard, nanosized materials, such as nanoparticles of titanium dioxide (TiO_2_), silver (Ag), and zinc oxide (ZnO), have been explored over the years to hinder microbial colonization on heritage substrate materials. Franco-Castillo et al. have recently published a comprehensive review about the use of nanoparticles as antimicrobial materials in the conservation of Cultural Heritage [94]. For applicative purposes, these nanosized active materials can be deposited over surfaces as dispersed in organic solvents or included in natural and synthetic polymers, to create a weathering-resistant coating. For instance, silver nanoparticles were mixed with a siloxane grafting agent to inhibit bacterial viability over *Serena* stones [95], while TiO_2_, Ag, and ZnO nanoparticles have been incorporated in various silane/siloxane-based polymeric matrices to grant protective and antifouling properties [96]. 

A different approach has been proposed by Presentato et al., who formulated a controlled-release system for the protection of stone artifacts from biological attack using mesoporous silica nanoparticles. These particles, synthesized by a sol-gel method assisted by *o*/*w* emulsion, have been loaded with Preventol RI-80, a commercial biocide, and then applied over degraded stone fragments. The biocide included in the mesoporous particles is then released over the surface in a gradual and controlled way, inhibiting bacterial growth over time [97]. Incorporation of engineered Ag/SiO_2_ nanoparticles in organically modified silica-based surface treatments was demonstrated to be an effective strategy to obtain anti-fouling coatings, thanks to the formation of a nano-roughness which leads to superhydrophobic behavior [98,99].

**Figure 8:**
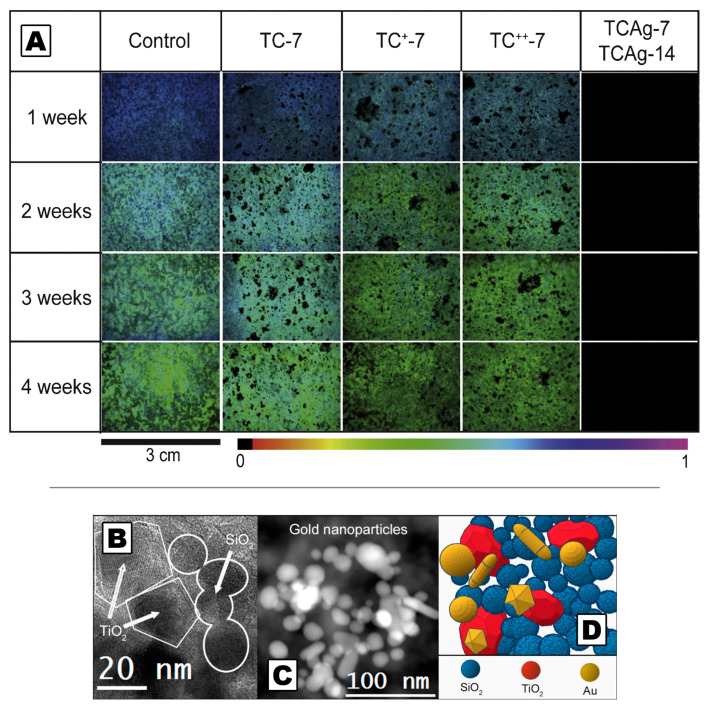
Fluorescence imaging of the dynamic evolution of chlorella vulgaris inoculated in stone treated with different coatings based on silane/siloxane emulsions with chitosan and AgNO_3_. Samples labeled as “Control” have not been treated (**A**). High Resolution Transmission Electron Microscopy image of the TiO_2_/SiO_2_ coating obtained by a sol-gel route (**B**). High Angle Annular Dark Field Scanning Transmission Electron Microscopy image of AuNPs into the coating (**C**). Schematic representation of the nanostructure of the coating (**D**). (**A**) Adapted from Reference [100], Copyright (2018), with permission from Elsevier. (**C**,**D**) Adapted from Reference [103].

On the other hand, protective coatings with antimicrobial properties have been recently formulated by using silane/siloxane emulsions combined with chitosan, a “green” and renewable biopolymer, and loaded with silver nitrate (see Figure 8A) [100]. 

With the aim of preventing dust and particulate deposition on outdoor artifacts, protective coatings have been also designed to act as self-cleaning surfaces, usually taking advantage of the photoactivity of TiO_2_. For instance, TiO_2_ nanorods have been added to methacrylic-siloxane resins to manufacture a self-cleaning functional coating for the protection of porous carbonate stone artifacts [101]. TiO_2_ nanoparticles can be also encapsulated in bisphenol A-free epoxy-silica resins to obtain multifunctional stone conservation products with consolidating, hydrophobic, and biocidal properties. Interestingly, the addition of Ce-doped titania nanoparticles to the epoxy-silica resins resulted in coatings showing excellent homogeneity and hydrophobicity, with increased self-cleaning and antimicrobial properties as compared to undoped and titania-doped films [102]. The prevention of soil deposition on stone can be also triggered by the addition of gold nanoparticles in TiO_2_/SiO_2_ coatings obtained by a simple sol-gel route (see Figure 8B). Gold nanoparticles boost the photoactivity of TiO_2_ under solar radiation by promoting absorption in the visible range [103].

One of the issues that recently emerged in the conservation of building materials is the protection of historical surfaces from graffiti or, more in general, from writings and tags [104]. Superhydrophobic coatings, such as fluorinated polymer matrices enriched with SiO_2_ or montmorillonite nanoparticles [105,106], have great potential, even if, to be applied on Cultural Heritage items, they must be transparent, resistant to aging and completely removable, key features that are easily achieved. To overcome the limits of available anti-graffiti coatings, which are poorly stable and mainly based on fluorinated compounds, a new kind of smooth anti-fouling coatings based on methyltrimethoxysilane has been recently proposed [107]. These coatings possess high repellency to various fluids, as well as show high stability and high transparency.

### 4.2. Surface Protection of Metal Artifacts

The preservation of metal artifacts is threatened by corrosion processes that are triggered by the presence of outdoor and indoor pollutants. For instance, copper-based alloys, widely used since ancient times, are affected by the so-called “bronze disease” that leads to formation of atacamite and its polymorphs, which are mainly responsible for the transformation of the alloy in a greenish powder. Several approaches have been recently explored to hamper or slow down corrosion. Green and renewable matrices have been used to confine and gradually release corrosion inhibitors. For instance, imidazolium salts confined in chitosan-based coatings have been used to protect bronze surfaces [108]. In addition to that, similar chitosan-based coatings have been loaded with benzotriazole and mercaptobenzothiazole for the protection of indoor works of art containing copper-based alloys [109].

Owing to the known toxicity of benzotriazole derivatives, research efforts have been also devoted to the formulation of novel corrosion inhibitors, such as triazole thiones, which have been proved efficient in inhibiting both anodic and cathodic corrosion processes of bronze samples [110].

**Figure 9:**
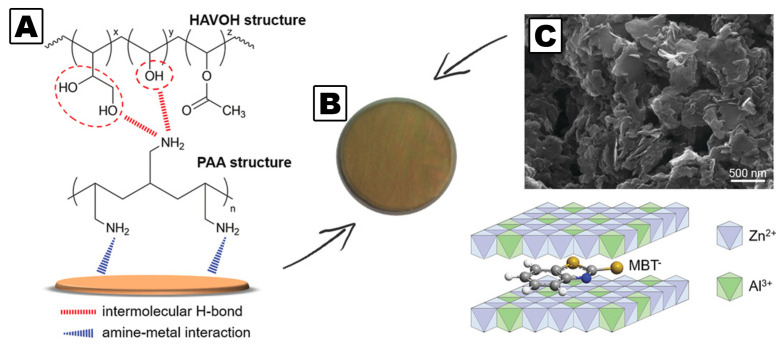
Schematic representation of the interactions between bronze substrates and amorphous polyvinyl alcohol and poly(allylamine), whose chemical structures are also shown (**A**). A bronze sample coated with the developed protective layer (**B**). Scanning electron microscopy image of layered double hydroxide/2-mercaptobenzothiazole system and the corresponding structure (**C**). Adapted from ref [111].

Environmentally friendly organic corrosion inhibitors containing at least a carboxylic moiety, such as p-aminobenzoic acid, have been used for the functionalization of both silica and layered double hydroxide nanoparticles, to create a reservoir of corrosion inhibitor on steel, released upon stimuli based on the chemical environment [111]. Active protective coatings have also been obtained using amorphous PVA loaded with 2-mercaptobenzothiazole incapsulated in layered double hydroxide nanocarriers. Besides its efficacy in inhibiting metal corrosion, the obtained coatings are transparent, a key feature for the protection of metals whose mirror-like finishing must not be altered by conservation treatments (see Figure 9) [112].

Finally, the protective ability of fluoroethylene/vinylether alternating copolymers coatings deposited on bronze surfaces has been recently increased by the addition of silsesquioxane nanoparticles, which enhance the plasticity and the abrasion resistance of the coating [113].

## 5. Perspectives

The formulation of advanced materials for art conservation is a vast topic where several challenges remain, and many possibilities can still be explored. Despite the diverse systems and applications discussed in this review, research in the field is far from concluded. One of the major trends in conservation science involves the growing formulation of solutions based on green chemistry, coping with the dictates of the EU Green Deal. There are several green solvents, among those reviewed in the literature [114], which might be selected for cleaning fluids or gelled systems, bearing in mind that the very definition of “green solvent” needs accuracy [115]. The aforementioned alkyl carbonates are optimal cleaning fluids, while some ionic liquids could be potentially explored in the next few years [116,117,118]. Following the same principles, bio- and natural materials are strongly encouraged as consolidants, gelling, or film-forming molecules. Chitosan and fibroin are two major examples, but many other natural sugars and proteins (e.g., of vegetal origin) can be considered, as well as the possibility of combining nano-scale fibers with sugar or protein films to achieve coatings with optimal properties. Natural compounds could also be used as the base for pollutants absorbers, so as to protect objects through preventive conservation in museums and storages. Life Cycle Assessment (LCA) of any developed formulation has become essential to evaluate and minimize the ecotoxicological impact of innovative solutions “from cradle to grave”.

Another key topic is the formulation of stimuli-responsive systems, a concept derived from drug-delivery. For instance, gels or coatings able to release active molecules in response to changes in pH have been proposed for the protection of metals, but the concept has general value. Changes in the rheological properties of gels/coatings in response to mechanical stimuli might be useful to improve the reversibility of treatments, or to adapt these systems to surfaces with different roughness.

Finally, resiliency is a key-concept. Materials for preserving works of art should be developed in such a way that their application leads to stable and durable treatments, decreasing maintenance costs and boosting the accessibility of the artifacts.

The number and type of conservation challenges remain vast. The concerning issue of climate changes and natural disasters endangers the conservation of outdoor artifacts, historical buildings, and archaeological sites. On the other hand, modern and contemporary art has often been (and still is) produced using industrial or disposable materials that are not conceived to last long. Paint layers or plastic surfaces can exhibit high sensitiveness to conventional solvents and cleaning fluids, or display severe mechanical failure owing to fast degradation processes. Overall, the call remains to materials scientists, to formulate new advanced materials for the safeguard of our Cultural Heritage.

## Data Availability

Not applicable.
